# Evaluating the associations between compliance with CKD guideline component metrics and renal outcomes

**DOI:** 10.1038/s41598-024-62152-6

**Published:** 2024-05-20

**Authors:** Zannatun Nyma, Kaori Kitaoka, Yuichiro Yano, Hiroshi Kanegae, Nomin Bayaraa, Seiji Kishi, Hajime Nagasu, Toshiaki Nakano, Jun Wada, Shoichi Maruyama, Naoki Nakagawa, Kouichi Tamura, Takashi Yokoo, Motoko Yanagita, Ichiei Narita, Kunihiro Yamagata, Takashi Wada, Kazuhiko Tsuruya, Naoki Nakashima, Yoshitaka Isaka, Masaomi Nangaku, Naoki Kashihara, Hirokazu Okada, J-CKD-DB study collaborative, Yoshio Terada, Shin-ichi Araki, Masanori Emoto, Yusuke Suzuki, Kazuhiko Ohe, Mihoko Okada, Eiichiro Kanda, Hiromi Kataoka

**Affiliations:** 1 Noncommunicable Disease (NCD) Epidemiology Research Center, Shiga University of Medical Science, Otsu, Japan; 2 Department of General Medicine, Faculty of Medicine, Juntendo University, Tokyo, Japan; 3 Department of Family Medicine and Community Health, Duke University, Durham, NC USA; 4 Office of Research and Analysis, Genki Plaza Medical Center for Health Care, Tokyo, Japan; 5 Department of Nephrology and Hypertension, Kawasaki Medical School, Kurashiki, Japan; 6 Department of Medicine and Clinical Science, Graduate School of Medical Sciences, Kyushu University, Fukuoka, Japan; 7 Department of Nephrology, Rheumatology, Endocrinology and Metabolism, Okayama University Graduate School of Medicine, Dentistry and Pharmaceutical Sciences, Okayama, Japan; 8 Department of Nephrology, Nagoya University Graduate School of Medicine, Nagoya, Japan; 9 Division of Cardiology and Nephrology, Department of Internal Medicine, Asahikawa Medical University, Asahikawa, Japan; 10 Department of Medical Science and Cardiorenal Medicine, Yokohama City University Graduate School of Medicine, Yokohama, Japan; 11 Division of Nephrology and Hypertension, Department of Internal Medicine, The Jikei University School of Medicine, Tokyo, Japan; 12 Department of Nephrology, Graduate School of Medicine, Kyoto University, Kyoto, Japan; 13 Division of Clinical Nephrology and Rheumatology, Niigata University Graduate School of Medical and Dental Sciences, Niigata, Japan; 14 Department of Nephrology, Faculty of Medicine, University of Tsukuba, Tsukuba, Japan; 15 Department of Nephrology and Laboratory Medicine, Kanazawa University, Kanazawa, Japan; 16 Department of Nephrology, Nara Medical University, Kashihara, Japan; 17 Department of Medical Informatics, Graduate School of Medicine, Kyushu University, Fukuoka, Japan; 18 Department of Nephrology, Osaka University Graduate School of Medicine, Suita, Japan; 19 Division of Nephrology and Endocrinology, the University of Tokyo Graduate School of Medicine, Tokyo, Japan; 20 Kawasaki Medical School, Kawasaki Geriatric Medical Center, Okayama, Japan; 21 Department of Nephrology, Faculty of Medicine, Saitama Medical University, 38 Moro-Hongo, Moroyama-Machi, Iruma-Gun, Saitama, 350-0495 Japan; 22 Department of Endocrinology, Metabolism and Nephrology, Kochi Medical School, Kochi University, Kochi, Japan; 23 Division of Nephrology, Department of Internal Medicine, Wakayama Medical University, Wakayama, Japan; 24 Department of Nephrology, Osaka Metropolitan University Graduate School of Medicine, Osaka, Japan; 25 Department of Nephrology, Faculty of Medicine, Juntendo University, Tokyo, Japan; 26 Department of Biomedical Informatics, Graduate School of Medicine, The University of Tokyo, Tokyo, Japan; 27 Institute of Health Data Infrastructure for All, Tokyo, Japan; 28 Department of Medical Science, Kawasaki Medical School, Kurashiki, Japan; 29 Faculty of Health Science and Technology, Kawasaki University of Medical Welfare, Kurashiki, Japan

**Keywords:** CKD, Real-world clinical scenarios, Compliance to guidelines, Clinical questions, End-stage kidney disease, Chronic kidney disease, Medical research, Nephrology

## Abstract

Understanding the association between compliance to the Chronic Kidney Disease (CKD) guidelines in real-world clinical settings and renal outcomes remains a critical gap in knowledge. A comprehensive analysis was conducted using data from a national, multicenter CKD registry. This study included 4,455 patients with an estimated glomerular filtration rate (eGFR) measurement on the index date and eight additional metrics recorded within six months. These metrics comprised serum electrolyte levels, low-density lipoprotein cholesterol, hemoglobin, and the use of renin-angiotensin system inhibitors. The primary outcome was a composite of renal events, defined by a decline in eGFR to < 15 mL/min/1.73 m^2^ or a reduction of ≥ 30% in eGFR, confirmed by follow-up tests. Over a median follow-up of 513 days, 838 renal events were observed. High serum potassium levels (> 5.4 mmol/L) were associated with increased event rates compared to lower levels. Similarly, low serum sodium-chloride levels (< 33) correlated with higher event rates. Usage of renin-angiotensin system inhibitors, low serum calcium (< 8.4 mg/dL), and high uric acid levels (> 7.0 mg/dL) were also linked to increased events. Conversely, higher hemoglobin levels (≥ 13 g/dL) were associated with lower event rates. Compliance to guidelines, categorized into quartiles based on the number of met metrics, revealed a significantly reduced risk of events in the highest compliance group (meeting 8 metrics) compared to the lowest (0–5 metrics). Compliance to CKD guidelines in clinical practice is significantly associated with improved renal outcomes, emphasizing the need for guideline-concordant care in the management of CKD.

## Introduction

An emerging problem in both clinical and ambulatory medicine is chronic kidney disease (CKD)^1^. Approximately 13% of the Japanese adult population is estimated to have CKD^2^. CKD is known as not only a worldwide public health problem, but also a global socioeconomic concern. CKD encompasses many adverse outcomes including kidney failure, cardiovascular disease, and premature death^3^. Aging and lifestyle habits have an impact on kidney function. Japan is experiencing an increase in its elderly demographic, and it is projected that the incidence of CKD will rise correspondingly within this aging populace in the imminent future^4,5^. The number of end-stage kidney disease (ESKD) patients has also continued to increase in Japan^6,7^.

Clinical practice guidelines aid many purposes. Guidelines help clinicians and other caregivers deal with the exponential growth in medical literature, help to expose gaps in knowledge, and suggest areas where additional research is needed^8^. Professional societies throughout the world decided that there is a need for developing clinical practice guidelines for patients with CKD because there are variations in the treatment approaches across different regions, leading to inconsistent patient outcomes. The Evidence-based Clinical Practice Guidelines for CKD 2018 and its associated guidelines^9–11^ provides evidence-driven recommendations addressing clinical questions (CQ) related to CKD treatment in Japan. Yet, the actual compliance to that guideline in clinical settings within Japan remains unevaluated. Moreover, the association between this compliance rate and renal outcomes in CKD patients is undetermined. Identifying the association between compliance rates and renal outcomes in CKD patients is essential for advancing medical knowledge, improving patient care, informing healthcare policies, and ultimately enhancing the quality of life for individuals with CKD.

To bridge this research gap, our objective is to assess the degree of implementation of the guideline recommendations in real-world clinical scenarios and their subsequent associations with renal outcomes.

## Results

A cohort of 11,333 individuals was identified as potential study participants. These individuals had undergone measurements for eight distinct component metrics pertaining to clinical questions, in addition to estimated glomerular filtration rate (eGFR) assessments. Of the 11,333 individuals, 146 individuals with a baseline eGFR below 15 mL/min/1.73m^2^ were excluded. An additional 6,732 were removed due to lacking eGFR measurements after the index date, resulting in a final sample size of 4,455 individuals for the analysis. Participants included in this study were younger, had lower eGFR, were receiving renin-angiotensin system (RAS) inhibitors, and demonstrated reduced levels of calcium, phosphorus, and low-density lipoprotein (LDL) cholesterol at the index date e compared to those excluded from the analysis (Supplementary Table S1).

**Figure 1:**
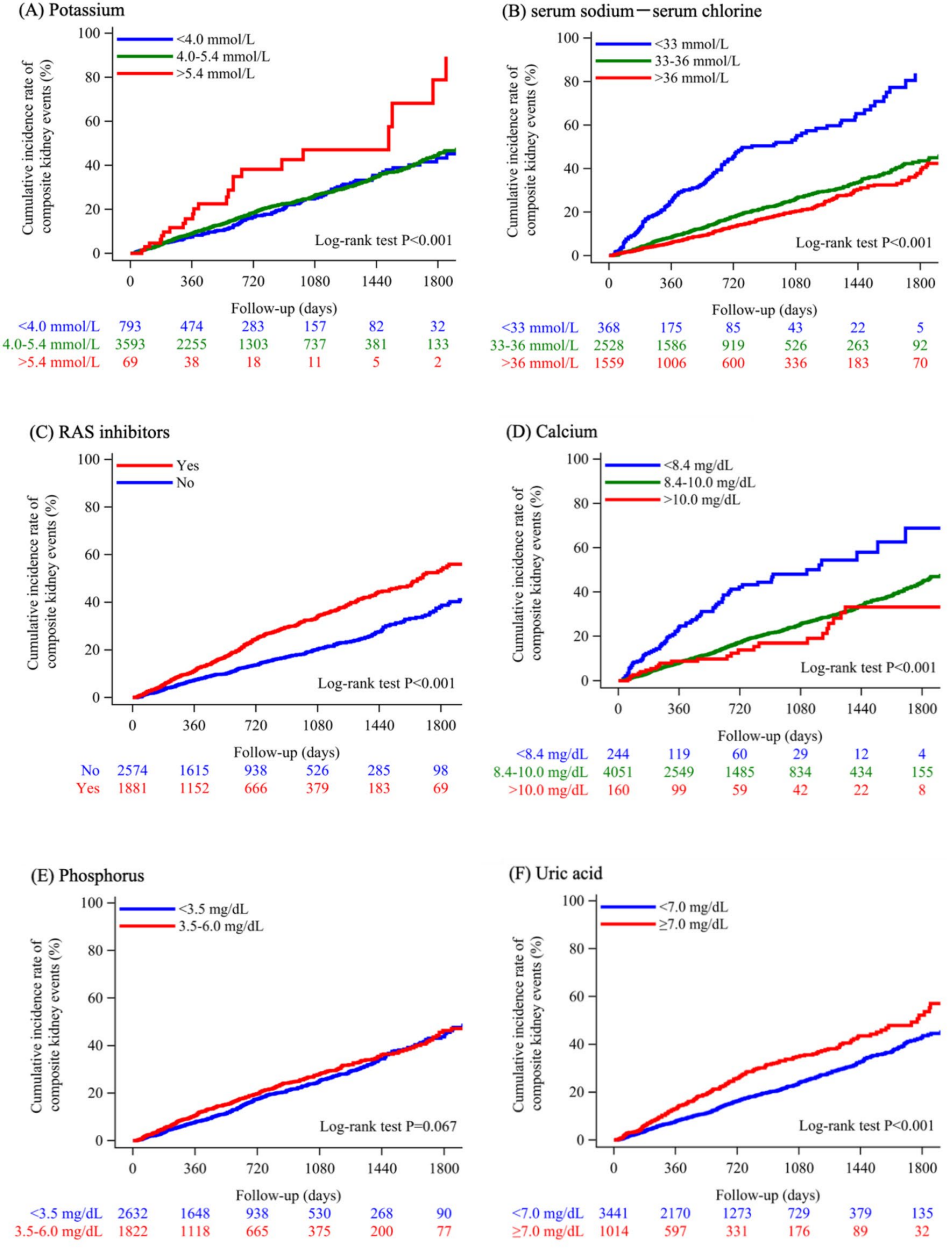

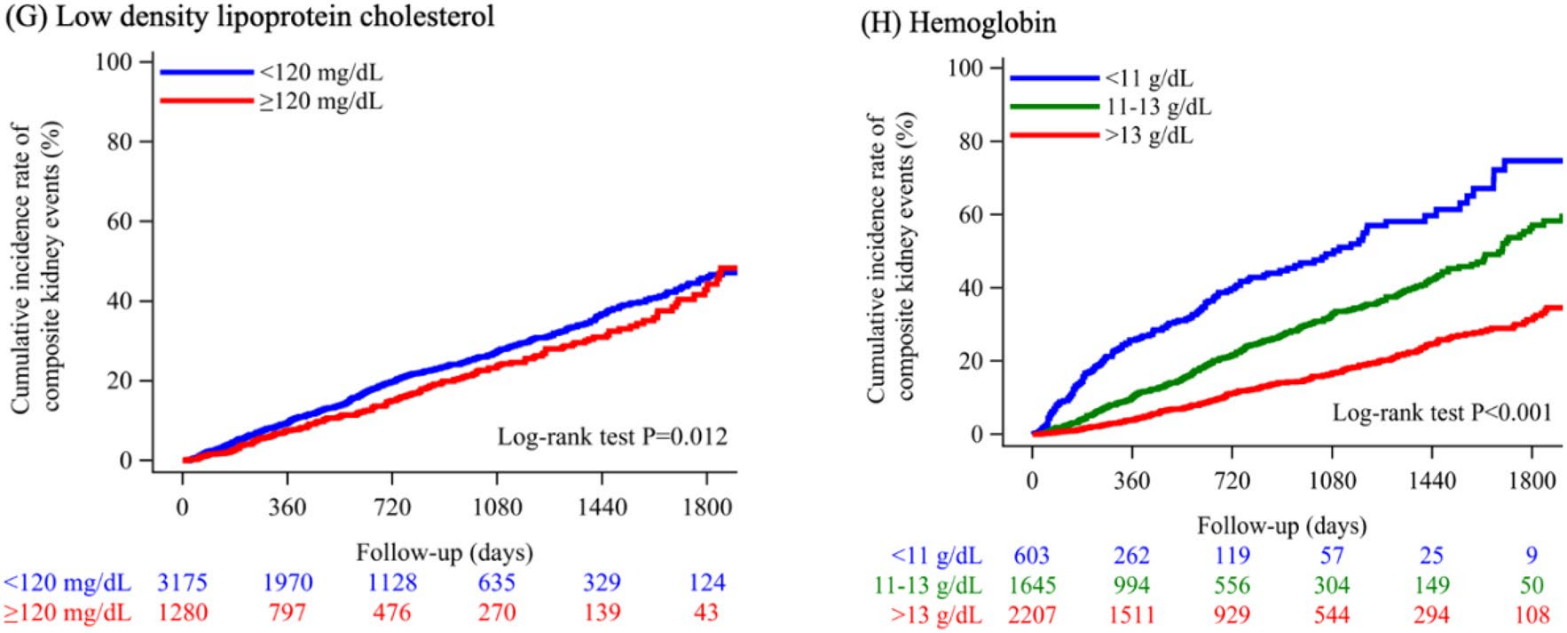
Cumulative incidence of composite renal events across groups of eight component metrics based on compliance to the 2018 CKD Clinical Practice Guidelines and its associated guidelines. We defined composite renal events as either the onset of ESKD, characterized by a decline in eGFR to less than 15 mL/min/1.73 m^2^, or a reduction of 30% or more in eGFR from the index date, with both conditions requiring confirmation by follow-up tests. To evaluate the disparities in the cumulative incidence of the composite renal events among different groups that were categorized based on the compliance to the 2018 CKD Clinical Practice Guidelines and its associated guidelines, we applied the Kaplan–Meier method. Furthermore, we utilized the log-rank test to determine the statistical significance, as indicated by the P-value. CKD = chronic kidney disease; eGFR = estimated glomerular filtration rate; ESKD = end-stage kidney disease; LDL cholesterol = low-density lipoprotein; RAS = renin-angiotensin system.

Table 1 details the baseline characteristics of the study participants: 53.5% were male, the average age was 67.2 years, and the mean eGFR was 54.6 mL/min/1.73 m^2^. Patients underwent a median of 8 eGFR measurements, with the interquartile range spanning from 4 to 15. The median interval between these assessments was 60 days, with the interquartile range spanning from 38 to 92 days. Figure 1 illustrates the cumulative incidence of renal events across groups based on compliance to the 2018 CKD Clinical Practice Guidelines and its associated guidelines as follows^9–11^. In Fig. 1A, those with serum potassium > 5.4 mmol/L had higher event rates than those < 4.0 mmol/L and those between 4.0–5.4 mmol/L. In Fig. 1B, groups with serum sodium-chlorine < 33 had higher rates than those between 33–36 and > 36. In Fig. 1C, groups taking RAS inhibitors had higher rates than those not taking them. In Fig. 1D, groups with serum calcium < 8.4 mg/dL had higher rates than those ≥ 8.4 mg/dL. In Fig. 1F, groups with serum uric acid ≥ 7.0 mg/dL had higher rates than those < 7.0 mg/dL. Lastly, in Fig. 1H, groups with hemoglobin < 11 g/dL had the highest rates, followed by 11-13 g/dL and ≥ 13 g/dL. Analysis of renal event rates shows no significant difference in the cumulative incidence when comparing groups with serum phosphorus at or below 6.0 mg/dL and those below 6 (Fig. 1E), and between the groups with LDL cholesterol levels below and at or above 120 mg/dL (Fig. 1G).

**Table 1:** Baseline clinical characteristics at the index date.

Variables	Overall, n = 4,455
Age, years	67.2 ± 14.0
Men, %	2384 (53.5)
eGFR, mL/min/1.73m^2^	54.6 ± 20.5
Potassium, mmol/L	4.3 ± 0.5
Sodium − Chlorine, mmol/L	35.6 ± 2.4
Use of RAS inhibitors, yes	1881 (42.2)
Calcium, mg/dL	9.1 ± 0.5
Phosphorus, mg/dL	3.3 ± 0.6
Uric acid, mg/dL	5.9 ± 1.5
Low-density lipoprotein cholesterol, mg/dL	104.3 ± 30.3
Hemoglobin, g/dL	13.0 ± 1.8
Hypertension, n (%)	3022 (67.8)
Diabetes mellitus, n (%)	3603 (80.9)
Glomerulonephritis, n (%)	895 (20.1)
Nephrotic syndrome, n (%)	581 (13.0)

Data are expressed as means (standard deviations) or percentages.

*eGFR* estimated glomerular filtration rate, *RAS* renin-angiotensin system.

Participants were categorized based on their compliance to the guidelines for each metric, distinguishing between those who complied and those who did not (Supplementary Table S2). During the median follow-up period of 513 days, with an interquartile range of 213 to 959 days, there were 838 composite renal events. In unadjusted models, participants who demonstrated compliance with the guidelines, specifically in managing serum potassium, sodium-chloride, calcium, urine acid, and hemoglobin levels experienced fewer composite kidney events compared to their non-compliant counterparts (Table 2). In adjusted analyses, which simultaneously accounted for all seven metrics, as well as age, sex, and eGFR at the index date, participants who demonstrated compliance with the guidelines, specifically in managing serum potassium, sodium-chloride, calcium, and hemoglobin levels experienced fewer composite kidney events compared to their non-compliant counterparts (Table 2). In the adjusted model, we assessed whether the association between compliance to CKD guidelines and composite renal events varies based on the participants' eGFR levels above versus below 45 mL/min/1.73 m^2^ at the index date. We identified a significant interaction (P = 0.01) based on eGFR levels at the index date, categorized as ≥ 45 mL/min/1.73 m^2^ versus < 45 mL/min/1.73 m^2^, in examining how compliance to LDL cholesterol guidelines was associated with renal event outcomes. Consequently, we conducted stratified analyses according to these eGFR categories at the index date. A difference in renal outcomes was evident between individuals with LDL cholesterol levels below 120 mg/dL and those with levels at or above this threshold, but this discrepancy was only apparent in the subgroup with eGFR levels below 45 mL/min/1.73 m^2^ at the index date (hazard ratios [HRs] 0.75, 95% confidence intervals [CIs] 0.60 to 0.94).

**Table 2:** Associations between compliance to each of the eight specific metrics outlined in the 2018 Evidence-Based Clinical Practice Guidelines for CKD and its associated guidelines and composite renal events.

Variables	Composite renal events	Unadjusted		Adjusted	
	Events/n	HRs (95% CIs)	*P* value	HRs (95% CIs)	*P* value
(A) Potassium					
> 5.4 mmol/L	23/69	1		1	
≤ 5.4 mmol/L	815/4386	0.46 (0.31, 0.70)	< 0.001	0.68 (0.44, 1.03)	0.067
(B) Sodium-Chlorine					
< 33 mmol/L	141/368	1		1	
≥ 33 mmol/L	697/4087	0.32 (0.27, 0.38)	< 0.001	0.57 (0.46, 0.69)	< 0.001
(C) Use of RAS inhibitors					
No	376/2574	1		1	
Yes	462/1881	1.73 (1.51, 1.98)	< 0.001	1.40 (1.21, 1.61)	< 0.001
(D) Calcium					
< 8.4 mg/dL	82/244	1		1	
≥ 8.4 mg/dL	756/4211	0.39 (0.31, 0.49)	< 0.001	0.56 (0.44, 0.71)	< 0.001
(E) Phosphorus					
> 6 mg/dL	0	-	-	-	-
≤ 6 mg/dL	838/4455				
(F) Uric acid					
≥ 7.0 mg/dL	244/1014	1		1	
< 7.0 mg/dL	594/3441	0.64 (0.56, 0.75)	< 0.001	0.85 (0.73, 1.00)	0.05
(G) Low-density lipoprotein cholesterol					
≥ 120 mg/dL	209/1280	1		1	
< 120 mg/dL	629/3175	1.22 (1.04, 1.43)	0.013	1.03 (0.88, 1.20)	0.748
(H) Hemoglobin					
< 11 g/dL	194/603	1		1	
≥ 11 g/dL	644/3852	0.32 (0.27, 0.38)	< 0.001	0.43 (0.36, 0.52)	< 0.001

Associations between adherence to each of the eight specific metrics outlined in the 2018 Evidence-Based Clinical Practice Guidelines for CKD and its associated guidelines and composite renal events in unadjusted and adjusted models. The median follow-up duration spanned 513 days and had an interquartile range from 213 to 959 days. Adjusted model included all eight metrics into the same model simultaneously, along with age, sex, and the estimated glomerular filtration rate at the index date.

*CKD* chronic kidney diseases, *RAS* renin-angiotensin system, *HRs* Hazard ratios, *CIs* confidence intervals.

**Figure 2:**
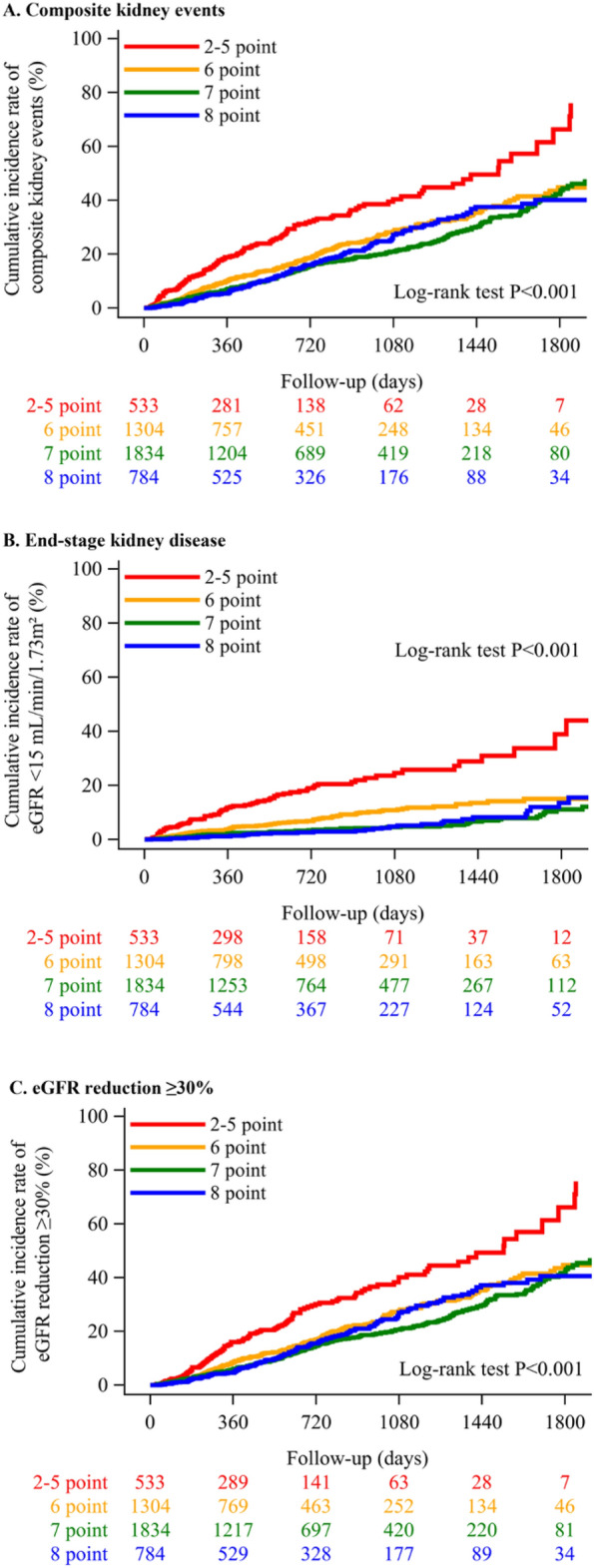
Cumulative incidence of composite renal events across groups based on compliance to the 2018 CKD Clinical Practice Guidelines and its associated guidelines. We defined composite renal events as either the onset of ESKD, characterized by a decline in eGFR to less than 15 mL/min/1.73 m^2^, or a reduction of 30% or more in eGFR from the index date, with both conditions requiring confirmation by follow-up tests. We employed the Kaplan–Meier method to assess variations in the cumulative incidence of these renal events among quartiles, which were stratified according to compliance to the metrics set forth in the 2018 CKD Clinical Practice Guidelines and its associated guidelines. The log-rank test was then used to ascertain statistical significance, denoted by the *P*-value. CKD = chronic kidney disease; eGFR = estimated glomerular filtration rate; ESKD = end-stage kidney disease.

Participants were categorized into quartiles based on their compliance to each metric, as detailed in Fig. S2. The quartiles were defined by scores of 0–5 points with n = 533 participants, 6 points with n = 1,304 participants, 7 points with n = 1,844 participants, and 8 points with n = 784 participants. During the follow-up period, 143 composite events, which included a decline in eGFR by ≥ 30% and ESKD, were recorded in the 0–5 point group. In contrast, the 8 point group witnessed 143 events, as detailed in Table 3. The 0–5 point group exhibited the highest cumulative incidence of composite events when compared to other quartiles (Fig. 2). In terms of the rate of composite events per 1,000 person-years, the 0–5 point group had the highest at 192, followed by the 6-point group at 110, the 7-point group at 90, and the 8-point group at 95. In an unadjusted model, the group with 6 points (HRs 0.55, 95% CIs 0.45 to 0.68), the group with 7 points (HRs 0.45, 95% CIs 0.37 to 0.54), and the group with 8 points (HRs 0.47, 95% CIs 0.38 to 0.60) had a significantly lower risk of composite events compared with the group with 0–5 points (Table 3). After multivariable adjustment, the HRs (95% CIs) for composite events were HRs 0.67 (95% CIs 0.54 to 0.83) for the group with 6 points, HRs 0.55 (95% CIs 0.45 to 0.67) for the group with 7 points, and HRs 0.55 (95% CIs 0.44 to 0.70) for the group with 8 points. The groups with 6, 7, and 8 points each had a statistically significant lower risk of a ≥ 30% decline in eGFR or ESKD when compared to the group with 0–5 points (Table 3).

**Table 3:** Associations of clinical quality compliance with renal events: frequency, incidence, and hazard ratios.

Outcome	Point	n	Events	Unadjusted		Adjusted	
				HRs (95% CIs)	*P* value	HRs (95% CIs)	*P* value
Composite renal events	0–5	533	143	1.0	–	1.0	–
	6	1,304	243	0.55 (0.45–0.68)	< 0.001	0.67 (0.54–0.83)	< 0.001
	7	1,844	309	0.45 (0.37–0.54)	< 0.001	0.55 (0.45–0.67)	< 0.001
	8	784	143	0.47 (0.38–0.60)	< 0.001	0.55 (0.44–0.70)	< 0.001
eGFR < 15 mL/min/1.73m^2^	0–5	533	85	1.0	–	1.0	–
	6	1,304	90	0.36 (0.26–0.48)	< 0.001	0.72 (0.53–0.97)	0.030
	7	1,844	72	0.18 (0.13–0.25)	< 0.001	0.43 (0.31–0.59)	< 0.001
	8	784	33	0.19 (0.13–0.28)	< 0.001	0.62 (0.41–0.95)	0.026
eGFR decline ≥ 30%	0–5	533	130	1.0	–	1.0	–
	6	1,304	228	0.57 (0.46–0.70)	< 0.001	0.65 (0.52–0.80)	< 0.001
	7	1,844	297	0.47 (0.38–0.58)	< 0.001	0.54 (0.44–0.67)	< 0.001
	8	784	141	0.51 (0.40–0.65)	< 0.001	0.57 (0.45–0.73)	< 0.001

Participants were categorized into quartiles based on their compliance to each metric. The quartiles were defined by scores of 0–5 points with n = 533 participants, 6 points with n = 1,304 participants, 7 points with n = 1,844 participants, and 8 points with n = 784 participants. Adjusted model includes adjustment for age, sex, and eGFR at the index date.

*eGFR* estimated glomerular filtration rate, *HRs* Hazard ratios, *CIs* confidence intervals.

## Discussions

Our study is the first to utilize real-world data in a large-scale analysis of outcomes, demonstrating that compliance to CKD guidelines positively influences renal health. This assessment supports the efficacy of multidisciplinary therapeutic strategies, reinforcing the critical role of guideline-aligned care in CKD management.

The findings from our study indicate associations between electrolyte imbalances and the rates of adverse renal events. Specifically, groups with serum potassium > 5.4 mmol/L had higher event rates than those < 4.0 mmol/L and those between 4.0–5.4 mmol/L. Groups with a combined serum sodium-chloride level of less than 33 had higher event rates compared to those with levels within the 33–36 range and above 36. Additionally, groups with serum calcium levels below 8.4 mg/dL experienced higher event rates than those with levels of 8.4 mg/dL or higher. Serum phosphorus levels were consistently below 6.0 mg/dL across the observed cases, as instances of serum phosphorus reaching 6 mg/dL or higher were not detected in this cohort. This absence suggests a patient population with well-controlled phosphorus levels, potentially due to effective dietary management or treatment compliance. These results are suggestive within the context of previous studies^12–16^, which have shown that electrolyte imbalances may have significant implications for renal outcomes. For instance, abnormal serum sodium and chloride levels are known to be associated with hypertension, cardiovascular events, and renal vasoconstriction^17^, which could potentially lead to a decline in renal function over time. Furthermore, the levels of serum sodium minus chlorine (mmol/L) serve as a potential clinical biomarker for metabolic acidosis^14,18^, a condition known to exacerbate renal decline. Nonetheless, it should be noted that assessing serum HCO3- beyond the simple difference between serum sodium and chloride levels would provide a more precise indication of metabolic acidosis. However, in the current study, we identified that 161 individuals (representing 3.6% of the total population) had recorded measurements of serum HCO3-. Given this, we recognized that the sample size is insufficient for robust statistical analysis of these points. Low serum calcium has been implicated in parathyroid hormone dysregulation and subsequent mineral bone disease, which could exacerbate kidney disease progression^16^. However, these associations do not imply causation. The observed electrolyte abnormalities could be a consequence of declining renal function rather than a cause. Renal impairment often leads to dysregulation of electrolyte homeostasis, and these electrolyte disturbances may serve as markers of disease severity rather than direct contributors to disease progression. Future prospective studies and clinical trials are needed to further elucidate the causative roles of these electrolyte imbalances in renal disease progression and to determine if targeted interventions can improve patient outcomes.

The observation that groups on RAS inhibitors had a higher rate of composite renal events than those not on these medications is unexpected, given the established renal protective benefits of RAS inhibitors^19–21^. Potential explanations for these findings might include the varied severity of CKD at baseline, the increased risk of hyperkalemia associated with RAS inhibitors, and potential drug interactions with RAS inhibitors. Additionally, the potential for confounding by indication should be considered. This term describes a scenario where the underlying reason for prescribing a specific treatment is directly related to the observed outcome. In this context, it is possible that patients with more progressive CKD, such as those exhibiting significant proteinuria, are more frequently prescribed RAS inhibitors. This could distort the study's results, creating an apparent association between these medications and poorer outcomes, when in reality, these drugs are being administered to patients with more severe disease presentations. A meticulous analysis that stratifies patients by CKD stage, accompanying health conditions, and proteinuria levels, while adjusting for confounding variables, is warranted to shed light on these results.

Our findings indicate that CKD patients with serum uric acid levels at or exceeding 7.0 mg/dL had higher rates of adverse renal events. This is consistent with prior studies that identifies hyperuricemia as an independent risk factor for ESRD in Japanese populations, even after adjusting for confounders such as proteinuria, hypertension, and dyslipidemia^22^. However, the literature also suggests that interventions to reduce uric acid levels may not necessarily translate to renal protection^23–25^. These may indicate that increased uric acid levels could be a marker of renal damage rather than a causative factor. It may reflect the reduced excretion by the kidneys rather than contributing directly to kidney disease progression.

The stratification of hemoglobin levels in the present study suggests a graded association with renal outcomes, where the lowest hemoglobin group (< 11 g/dL) experienced the highest rates of renal complications, followed by those within the 11–13 g/dL range, and finally the group with levels ≥ 13 g/dL. This gradation echoes the findings of earlier research^26–28^. Although prior studies suggest that anemia correction correlates with overall improved health outcomes, the direct benefit of elevating hemoglobin levels on renal outcomes remains ambiguous^29–31^. It implies that while anemia management is beneficial, there is no clear evidence that increasing hemoglobin levels beyond a certain threshold confers additional renal protection.

Our research examined the differences in the frequency of combined and specific renal events across different patient groups categorized by their CQ scores. The results indicated that patients with the lowest CQ scores, ranging from 0 to 5 points, were more likely to have a composite of renal events than those in the three higher scoring brackets (6–8 points). This trend was consistent when the health outcomes were specified as either a reduction of at least 30% in the eGFR or the onset of ESKD. However, important lifestyle factors, including smoking habits and nutritional intake, and the measurements of blood pressure levels were not included in this study. These elements could significantly impact kidney function over the course of the follow-up period, and their absence in our data should be noted as a limitation. The cutoffs for categorizing using CQ scores were arbitrary that could affect the validity and applicability of the results. Determining the optimal cutoff points for CQ scores that accurately reflect patient risk categories is crucial for the reliability of the study. Furthermore, it remains uncertain whether potential synergistic effects between different CQ items were considered. Synergistic effects occur when the combined impact of two or more factors is greater than the sum of their separate effects. In the context of CKD, certain risk factors may interact in a way that significantly increases the risk of adverse renal events. Not accounting for these may oversimplify the relationships between CQ scores and health outcomes. Addressing these limitations in future research could involve developing a more robust method for determining CQ score cutoffs, analyzing the interaction between different risk factors, and ensuring that the point system reflects the relative importance of each risk factor. Additionally, a broader and more diverse patient population could help to understand the generalizability of the findings.

The current study has limitations. First, from a pool of 11,333 individuals identified as potential participants, 6,732 lacked a follow-up eGFR measurement post-index date. A possible explanation for this could be referrals to external clinics or general practitioners, as suggested by instances where patients were directed to university hospitals for evaluation of undiagnosed kidney issues. After assessments, those deemed not to require ongoing follow-up for kidney diseases within the university hospital system were referred back to their initial healthcare providers. Unfortunately, the study does not provide specific reasons for the missing follow-up eGFR data for these 6,732 patients, which limiting insight into patient care continuity. Secondly, the study faces potential selection bias. The included participants were generally younger, had lower eGFR levels, were on RAS inhibitors, and exhibited lower levels of calcium, phosphorus, and LDL cholesterol at baseline compared to those excluded. This selection of participants may affect the generalizability and interpretation of our results. Third, this dataset is derived from real-world clinical settings, where physicians determined follow-up intervals based on individual patient needs and clinical judgments. As a result, the specific intervals between eGFR measurements varied widely, which potentially affecting the generalizability and interpretation of our results. Fourth, in the current study, non-compliance among participants included both individuals receiving medication and those not receiving medication. Consequently, the potential effect of medication use on our findings was not assessed, leaving its effects uncertain.

Our study revealed that compliance to CKD guideline recommendations in clinical practice correlates with renal outcomes, underscoring the importance of guideline-concordant care, especially in a multidisciplinary fashion, in managing CKD.

## Methods

The Japan Chronic Kidney Disease Database (J-CKD-DB) is a real-world electronic health record-based registry of CKD patients^32,33^. J-CKD-DB-Ex was developed based on J-CKD-DB system as a longitudinal CKD database. Data were taken from 4 university hospitals in Japan and data collection were started in January 2014, the current study is using the date followed until December 31st, 2020. This database incorporates information on inpatient and outpatient encounters, prescriptions, diagnostic codes, and laboratory measurements. The facilities participating in J-CKD-DB-Ex were required to have electronic health record systems that incorporated Standardized Structured Medical Information exchange 2 (SS-MIX2) (https://www.ss-mix.org/consE/) storage. The facilities were also required a structured data entry function that could transfer the data to the SS-MIX2 storage system^34^. With the use of SS-MIX2 storage, all data elements were extracted automatically for the avoidance of input error. After extraction, data were sent to the J-CKD-DB-Ex data center.

Fundamental standards were adopted in SS-MIX2 regarding patient profiles (the Health Level Seven [HL7] V2.5 [ISO 27931] data format), prescriptions (national drug code in Japan, HOT code), laboratory test results (Japan Laboratory Code Version 10 [JLAC10] code), diagnoses (ICD-10), and incidence of major outcomes^35–37^.

This study was conducted under the oversight of the Ethical Committee of the Saitama Medical University (2022–036) and in accordance with the principles of the Declaration of Helsinki. Informed consent was obtained through an opt-out method on the website of each participating university hospital in accordance with the Ethical Guidelines for Medical and Health Research Involving Human Subjects in Japan. The inclusion criteria for the J-CKD-DB-Ex were patients aged ≥ 18 years and patients with proteinuria ≥ 1 (dipstick test), or eGFR < 60 mL/min/1.73 m^2^ ^32^.

For the current analyses, we selected records of CKD patients leveraging the criteria of having an eGFR measurement documented on the index date (i.e., a starting point for the current study) alongside data on eight predetermined variables recorded within a six-month window preceding the index date (Fig. S1). These variables included serum potassium, serum sodium, serum chloride, serum calcium, serum phosphorus, uric acid, LDL cholesterol, and hemoglobin levels, in addition to the utilization of RAS inhibitors. The selection of these variables was grounded on the clinical advisements stipulated in the Evidence-based Clinical Practice Guidelines for CKD 2018 delineated by Japanese medical experts and by the data accessibility in the J-CKD-DB repository. We further confined our selection to CKD patient records showcasing at minimum, a single eGFR measurement transpiring after the index date.

### CKD guideline component metrics

We categorized the eight component metrics derived from the Evidence-Based Clinical Practice Guidelines for CKD 2018 and its associated guidelines as follows^9–11^: (A) serum potassium (mmol/L): < 4.0, 4.0–5.4, > 5.4; (B) serum sodium—chlorine (mmol/L): < 33, 33–36, > 36; (C) administration of RAS inhibitors (i.e., angiotensin converting enzyme inhibitors and angiotensin receptor blockers): yes or no; (D) serum calcium (mg/dL): < 8.4, 8.4–10.0, > 10.0; (E) serum phosphorus (mg/dL): < 3.5, 3.5–6.0, ≥ 6 ; (F) serum uric acid (mg/dL): < 7.0, ≥ 7.0; (G) LDL cholesterol (mg/dL): < 120, ≥ 120; (H) hemoglobin (g/dL): < 11, 11–13, > 13. Based on these categories, we developed a CQ recommendation scoring system as outlined in the Evidence-Based Clinical Practice Guidelines for CKD 2018 and its associated guidelines^9–11^ (see Supplementary Table 2), assigning one point for each of the following conditions being met: (A) serum potassium ≤ 5.4 mmol/L; (B) serum sodium – chlorine ≥ 33 mmol/L; (C) administration of RAS inhibitors: yes; (D) serum calcium ≥ 8.4 mg/dL; (E) serum phosphorus reaching 6 mg/dL or higher were not detected in this cohort. Therefore, serum phosphorus < 3.5 mg/dL was defined as the reference; (F) serum uric acid < 7.0 mg/dL; (G) LDL cholesterol < 120 mg/dL; (H) hemoglobin ≥ 11 g/dL, and quantifying compliance to each metric (Table S2) on a scale from 0 (poor compliance) to 8 (full compliance).

### Kidney function

Serum creatinine was assayed with an enzymatic method. eGFR was derived with the Chronic Kidney Disease Epidemiology Collaboration (CKD-EPI) equation modified by a Japanese coefficient^38^. The underlying causes of CKD were classified according to the International Classification of Diseases, Tenth Revision (ICD-10) as follows: 1) Glomerulonephritis (M321, N009, N014, N017, N019, N028-N030, N032, N033, N039); and 2) Nephrotic syndrome (N040, N042, N048, N049, N051-N055, N057, N059, N069, N083, N085). Additionally, participants were categorized based on the presence of hypertension, using ICD-10 codes I10, I110, I119, I120, I129, I139, I150-I152, I158, I159, and diabetes mellitus, identified through codes E100-E117, E119-E137, E139-E145, E148, E149, H280, H360, O240.

The primary outcome was the incidence of composite renal events across groups, classified based on their compliance to the metrics outlined in the Evidence-Based Clinical Practice Guidelines for CKD 2018^11^. We defined the composite renal evens as a persistent the onset of ESKD which was defined as an eGFR of < 15 mL/min/1.73 m^2^ (confirmed by a subsequent measurement), or reduction of 30% or more in eGFR (confirmed by a subsequent measurement). In the composite end point analyses, if a participant had more than one event occur, the first event was counted as the outcome. For example, if a participant who first undergoes a reduction in eGFR of 30% or more and then, a month later, is diagnosed with ESKD, we would count only the eGFR decline of 30% or more as the outcome in the composite endpoint analysis. However, in the endpoint-specific analyses, we would count both the eGFR decline of 30% or more and the ESKD diagnosis as separate outcomes.

### Statistical analysis

The descriptive statistics are presented as mean values and corresponding standard deviations, with proportions provided where relevant. We used to estimate survival functions for the Kaplan–Meier method, and compared to the differences between groups by the Log-rank test. Utilizing Cox proportional hazards regression models, we calculated HRs and their corresponding 95% CIs to assess the risk of renal events in relation to compliance or non-compliance to individual CKD guideline metrics. HRs were calculated in an unadjusted model (model 1), and after adjustments for covariates, including age, sex, and eGFR at the index date.

To determine whether the association between compliance to CKD guidelines and the incidence of composite renal events varies among the different stages of CKD, we assessed whether the association between compliance to CKD guidelines and composite renal events varies based on the participants' eGFR levels above versus below 45 mL/min/1.73 m^2^ at the index date. First, we assessed we tested for heterogeneity in the association between compliance to CKD guidelines and the incidence of composite renal events by eGFR at the index date (≥ 45 mL/min/1.73 m^2^ versus < 45 mL/min/1.73 m^2^) with the inclusion of multiplicative interaction terms. When we identified a significant interaction (P < 0.05), stratified analyses according to these eGFR categories at the index date were considered. The follow-up duration was established as the time from the index date to the earliest of the following: (1) patient's departure from the medical practice or database; or (2) the final date of data collection. Statistical significance was defined as a *P* value less than 0.05 using two-sided tests using SAS version 9.4 software (SAS Institute, Cary, NC).

### Supplementary Information

Supplementary Information.

## Data Availability

The data underlying this article will be shared on reasonable request to the corresponding author.

## Abbreviations

CKD: Chronic kidney disease
CKD-EPI: Chronic kidney disease epidemiology collaboration
CIs: Confidence intervals
CQ: Clinical questions
eGFR: Estimated glomerular filtration rate
ESKD: End-stage kidney disease
HRs: Hazar ratios
ICD-10: International Classification of Diseases, Tenth Revision
J-CKD-DB: Japan Chronic Kidney Disease Database
LDL cholesterol: Low-density lipoprotein cholesterol
RAS inhibitor: Renin-angiotensin system inhibitor
SS-MIX2: Standardized Structured Medical Information Exchange 2
