# Supplementary material for: Evaluating the associations between compliance with CKD guideline component metrics and renal outcomes
Source: Sci Rep. 2024 May 20;14:11481. doi: 10.1038/s41598-024-62152-6 (PMC11106300; doi:10.1038/s41598-024-62152-6)
Supplement: Supplementary file 1 — Supplementary Information. [file 41598_2024_62152_MOESM1_ESM.pdf]

Supplementary Material

Evaluating the Associations Between Compliance with CKD Guideline Component Metrics and Renal Outcomes

Zannatun Nyma; [1] Kaori Kitaoka;[1] Yuichiro Yano;[1-3] Hiroshi Kanegae;[4] Nomin Bayaraa;[1] Seiji Kishi;[5] Hajime Nagasu;[5] Toshiaki Nakano;[6] Jun Wada;[7] Shoichi Maruyama;[8] Naoki Nakagawa;[9] Kouichi Tamura;[10] Takashi Yokoo;[11] Motoko Yanagita;[12] Ichiei Narita;[13] Kunihiro Yamagata;[14] Takashi Wada;[15] Kazuhiko Tsuruya;[16] Naoki Nakashima;[17] Yoshitaka Isaka;[18] Masaomi Nangaku;[19] Naoki Kashihara; [20] and Hirokazu Okada;[21] J-CKD-DB study collaborative group; *

1. Noncommunicable Disease (NCD) Epidemiology Research Center, Shiga University of Medical Science, Shiga, Japan.
2. Department of General Medicine, Juntendo University Faculty of Medicine, Tokyo, Japan.
3. Department of Family Medicine and Community Health, Duke University, NC, USA.
4. Office of Research and Analysis, Genki Plaza Medical Center for Health Care, Tokyo, Japan.
5. Department of Nephrology and Hypertension, Kawasaki Medical School, Okayama, Japan.
6. Department of Medicine and Clinical Science, Graduate School of Medical Sciences, Kyushu University, Fukuoka, Japan.

7. Department of Nephrology, Rheumatology, Endocrinology and Metabolism, Okayama University Graduate School of Medicine, Dentistry and Pharmaceutical Sciences, Okayama, Japan.
8. Department of Nephrology, Nagoya University Graduate School of Medicine, Aichi, Japan.
9. Division of Cardiology and Nephrology, Department of Internal Medicine, Asahikawa Medical University, Hokkaido, Japan
10. Department of Medical Science and Cardiorenal Medicine, Yokohama City University Graduate School of Medicine, Yokohama, Japan.
11. Division of Nephrology and Hypertension, Department of Internal Medicine, The Jikei University School of Medicine, Tokyo, Japan.
12. Department of Nephrology, Graduate School of Medicine, Kyoto University, Kyoto, Japan.
13. Division of Clinical Nephrology and Rheumatology, Niigata University Graduate School of Medical and Dental Sciences, Niigata, Japan.
14. Department of Nephrology, Faculty of Medicine, University of Tsukuba, Ibaraki, Japan.
15. Department of Nephrology and Laboratory Medicine, Kanazawa University, Kanazawa, Japan.
16. Department of Nephrology, Nara Medical University, Nara, Japan.
17. Kyushu Universtity Hosipital, Medical Information Center, Fukuoka, Japan.
18. Department of Nephrology, Osaka University Graduate School of Medicine, Osaka, Japan.
19. Division of Nephrology and Endocrinology, the University of Tokyo Graduate

School of Medicine, Tokyo, Japan.
20. Kawasaki Geriatric Medical Center, Kawasaki Medical School, Okayama, Japan.
21. Department of Nephrology, Faculty of Medicine, Saitama Medical University, Saitama, Japan.
* Kawasaki Geriatric Medical Center, Kawasaki Medical School, Okayama, Japan.

Corresponding author:

Hirokazu Okada, MD, PhD,

Department of Nephrology, Faculty of Medicine, Saitama Medical University

ZIP: 38 Moro-hongo, Moroyama-machi, Iruma-gun, Saitama, 350-0495, JAPAN

Tel: +81- 49-276-1611

Email: hirookda@saitama-med.ac.jp

**Supplementary Table S1. Characteristics of participants who were included in the current study and those who were not included**			
**Variables**	Included (n=4,455)	Excluded (n=6,732)	p value
Age, years	67.2±14.0	68.3±13.3	<0.001
Men, %	2384 (53.5)	3572 (53.1)	0.642
eGFR, mL/min/1.73m2	54.6±20.5	56.8±19.1	<0.001
Potassium, mmol/L	4.34±0.46	4.31±0.48	0.002
Sodium−Chlorine, mmol/L	35.6±2.4	35.9±2.5	<0.001
Use of RAS inhibitors, yes	1881 (42.2)	2704 (40.2)	0.031
Calcium, mg/dL	9.1±0.5	9.3±0.6	<0.001
Phosphorus, mg/dL	3.3±0.6	3.4±0.6	<0.001
Uric acid, mg/dL	5.9±1.5	5.9±1.6	0.109
Low-density lipoprotein cholesterol, mg/dL	104.3±30.3	110.2±37.0	<0.001
Hemoglobin, g/dL	13.0±1.8	13.0±2.0	0.241
Data are expressed as means (standard deviation) or numbers (percentage). P values were calculated by unpaired t-test or chi-square test. eGFR=estimated glomerular filtration rate; RAS=renin-angiotensin system.			

Supplementary Table S2 Clinical questions scoring from the 2018 CKD Clinical Practice Guidelines

**Variables**	**1 point**	**0 point**
(A) Potassium (mmol/L)	≤5.4	>5.4
(B) Sodium-Chlorine (mmol/L)	≥33	<33
(C) Use of RAS inhibitors	Yes	No
(D) Calcium (mg/dL)	≥8.4	<8.4
(E) Phosphorus (mg/dL)	≤6.0	>6.0
(F) Uric acid (mg/dL)	<7.0	≥7.0
(G) Low-density lipoprotein cholesterol (mg/dL)	<120	≥120
(H) Hemoglobin (g/dL)	≥11	<11

Our evaluation of compliance to each metric was cumulative, with one point added for each criterion met, starting at 0 points to signify noncompliance and scaling up to a maximum of 8 points to represent complete, unyielding compliance. CKD=chronic kidney disease; RAS= renin-angiotensin system

Supplementary Figure S1

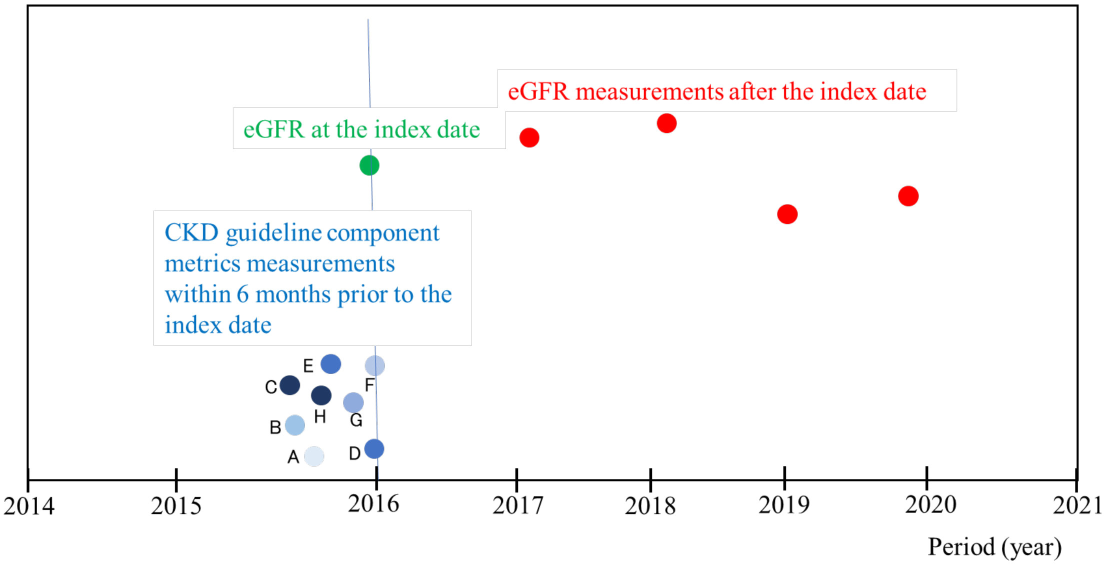

Supplementary Figure S2

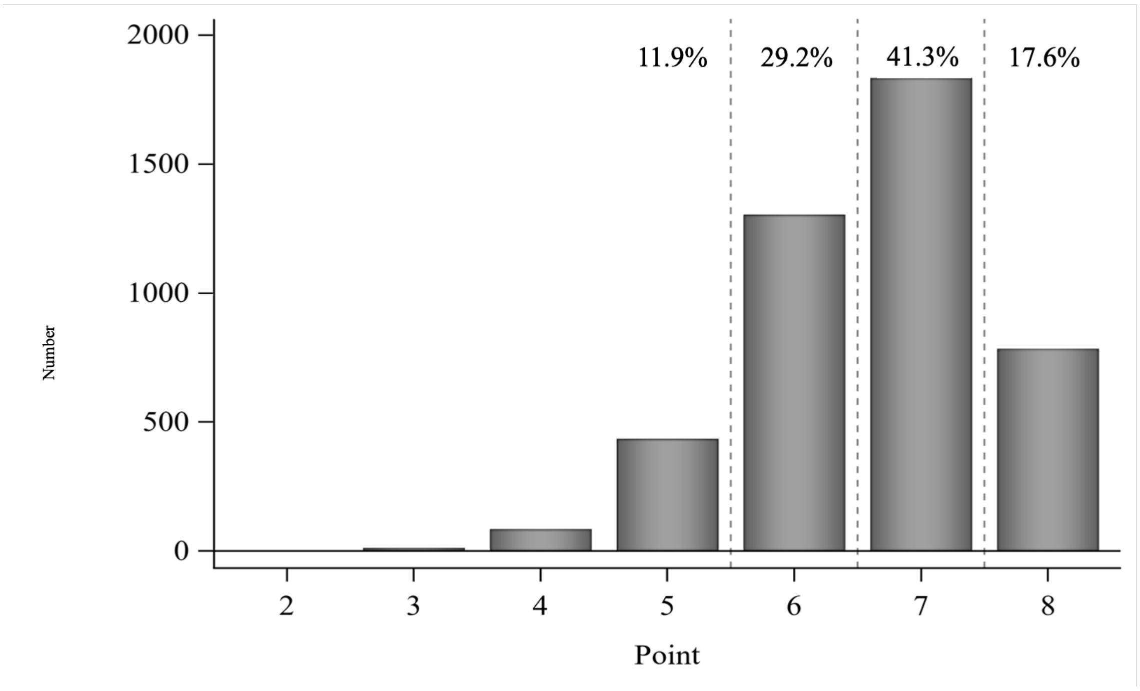

Figure legends

Supplementary Figure S1: Study design parameters and observational timeframe

we selected records of CKD patients leveraging the criteria of having an eGFR measurement documented on the index date (i.e., a starting point for the current study) alongside data on eight predetermined variables recorded within a six-month window preceding the index date (Figure S1). These variables included (A) serum potassium, (B) serum sodium and chloride, (C) serum calcium, (D) serum phosphorus, (E) uric acid, (F) LDL cholesterol, and (G) hemoglobin levels, in addition to (H) the utilization of RAS inhibitors. The selection of these variables was grounded on the clinical advisements stipulated in the Evidence-based Clinical Practice Guidelines for CKD 2018 and its associated guideline delineated by Japanese medical experts and by the data accessibility in the J-CKD-DB repository. We further confined our selection to CKD patient records showcasing at minimum, a single eGFR measurement transpiring after the index date.

CKD=chronic kidney disease; eGFR=estimated glomerular filtration rate; LDL=low-density lipoprotein; RAS=renin-angiotensin system.

Supplementary Figure S2: The distribution of scores of CQ recommendation and categorization of the quartiles based on their scores

We analyzed the distribution of CQ recommendation scores and classified them into quartiles, each defined by the range of scores it encompasses. Scores of CQ recommendation of 0-5 points group was 11.9%, 6 points group was 29.2%, 7 points group was 41.3%, 8 points group was 17.6%. CQ=clinical quality.

J-CKD-DB study collaborative group

Yoshio Terada;[22] Shin-ichi Araki;[23] Masanori Emoto;[24] Yusuke Suzuki;[25] Kazuhiko Ohe;[26] Mihoko Okada;[27] Eiichiro Kanda;[28] Hiromi Kataoka;[29]

22. Department of Endocrinology, Metabolism and Nephrology, Kochi Medical School, Kochi University, Kochi, Japan
23. Division of Nephrology, Department of Internal Medicine, Wakayama Medical University, Wakayama, Japan
24. Department of Nephrology, Osaka Metropolitan University Graduate School of Medicine, Osaka, Japan
25. Department of Nephrology, Juntendo University Faculty of Medicine, Tokyo, Japan
26. Department of Biomedical Informatics, Graduate School of Medicine, The University of Tokyo, Tokyo, Japan
27. Institute of Health Data Infrastructure for All, Tokyo, Japan
28. Department of Medical Science, Kawasaki Medical School, Kurashiki, Japan.
29. Faculty of Health Science and Technology, Kawasaki University of Medical Welfare, Kurashiki, Japan
